# A pilot study of a gratitude journaling intervention to enhance spiritual well-being and exercise self-efficacy in Black breast cancer survivors

**DOI:** 10.1186/s12888-024-06362-2

**Published:** 2024-12-18

**Authors:** Lakeshia Cousin, Dejana Braithwaite, Stephen Anton, Zhongyue Zhang, Ji-Hyun Lee, Christiaan Leewenburgh, Debra Lyon

**Affiliations:** 1 College of Nursing, University of Florida, Gainesville, USA; 2 Department of Surgery and Epidemiology, University of Florida, Gainesville, USA; 3 Department of Physiology and Aging, University of Florida, Gainesville, USA; 4 Department of Biostatistics, University of Florida, Gainesville, FL USA; 5 Biostatistics and Computational Biology Shared Resource, University of Florida Health Cancer Center, Gainesville, USA

**Keywords:** Breast cancer survivors, Health disparities, Spiritual well-being, Positive psychology, Randomized control trial

## Abstract

**Background:**

Breast cancer (BC) survivorship presents significant health disparities, particularly affecting Black women, who experience a 40% higher BC death rate compared to White women. These disparities are exacerbated by comorbidities, which contribute to poorer overall health outcomes. Additionally, Black BC survivors often face psychosocial challenges, including increased stress and lower well-being, which can lead to adverse physical health effects. This pilot study aims to assess the feasibility and efficacy of a culturally sensitive gratitude journaling intervention designed to enhance spiritual well-being, exercise self-efficacy, and reduce inflammation among Black BC survivors.

**Methods:**

This pilot study employed a two-group, parallel random-assignment experimental design to compare a gratitude journaling intervention with a general memory journaling control group. Twenty-six Black women aged 40 to 70 years with a history of BC were randomly assigned to either the gratitude journaling intervention group (*n* = 13) or the control group (*n* = 13). The gratitude intervention group engaged in gratitude journaling twice weekly for eight weeks, while the control group documented daily memories. Outcomes measured included Gratitude Questionnaire-6, FACIT-Spiritual Well-Being 12 Item Scale, Perceived Stress Scale, Giscombe Superwoman Schema Questionnaire, and the Stage of Motivational Readiness for Physical Activity questionnaire and inflammatory biomarkers. Statistical analyses included the Wilcoxon rank sum test and Fisher’s exact test.

**Results:**

Twenty-six participants were enrolled, with 73% completing baseline and post-intervention assessments. The intervention group showed a significant improvement in spiritual well-being (*p* = 0.014) with a large effect size (ES = 0.57). Marginal improvements in exercise self-efficacy were also observed (ES = 0.39). Although there were no significant differences in dispositional gratitude and perceived stress between groups, the intervention group exhibited trends toward increased gratitude and reduced stress. Inflammatory biomarker analysis indicated non-significant changes, though IL-6 levels increased in the intervention group.

**Conclusion:**

This study demonstrates the feasibility and acceptability of a gratitude journaling intervention among Black BC survivors. The intervention significantly enhanced spiritual well-being and showed promise in improving exercise self-efficacy, suggesting its potential for promoting holistic wellness in this population. These findings provide a foundation for future larger-scale randomized controlled trials to further evaluate the efficacy of gratitude-based interventions for Black BC survivors.

**Trial registration:**

This study was registered prospectively at ClinicalTrials.gov (NCT05473026) on 07-13-2022.

**Supplementary Information:**

The online version contains supplementary material available at 10.1186/s12888-024-06362-2.

## Background

Breast cancer (BC) survivorship care remains a significant health concern after treatment. Survivors often face adverse psychosocial and physical effects, including elevated stress levels, chronic inflammation, and poor lifestyle behaviors, all of which negatively impact their well-being and health-related quality of life [1–3]. According to the American Cancer Society [4], Black women still have a 4% lower incidence rate of breast cancer than White women but a 40% higher BC death rate. Moreover, a significant proportion of BC survivors face challenges related to comorbidities, which exacerbate their overall physical health outcomes [5, 6]. Among these comorbidities, metabolic syndrome—a cluster of conditions that increase the risk for cardiovascular disease and Type 2 diabetes—is particularly prevalent among Black women with BC [7, 8]. Metabolic syndrome not only amplifies the risk of developing BC but also contributes to poorer prognosis and outcomes, thus exacerbating existing health disparities among racial groups.

The intersection of race, age, and a cancer diagnosis creates unique challenges for Black women with breast cancer. Cultural constructs, such as the Superwoman Schema, which is deeply rooted in Black culture, further complicate the health landscape. This schema involves cognitive, affective, and behavioral responses emphasizing strength, emotional suppression, resistance to vulnerability, success despite adversity, and prioritizing others’ needs over one’s own. While these traits may foster resilience, they can also lead to heightened stress levels and increased vulnerability to metabolic syndrome and related health complications [9].

Promoting spiritual well-being among breast cancer survivors, particularly Black women, is critical not only for emotional resilience but also for reducing physiological risk factors associated with metabolic syndrome. Existing literature indicates a significant association between spiritual well-being and positive psychological constructs, such as gratitude, which serve as protective factors against metabolic syndrome [10–12]. Spiritual practices, including gratitude, meditation, and journaling, have been linked to improved metabolic parameters, such as blood pressure, blood glucose levels, and lipid profiles [13–16].

Cultivating dispositional gratitude—a trait linked to enhanced spiritual well-being, reduced psychological distress, and improved self-efficacy—offers a promising avenue for addressing these challenges [17–19]. According to Fredrickson’s Broaden-and-Build theory [20], gratitude broadens an individual’s cognitive and emotional resources, strengthening self-efficacy and resilience. Research on gratitude journaling pioneered by Emmons and colleagues supports this potential, demonstrating that gratitude interventions significantly improve well-being compared to neutral control conditions where participants simply documented general daily events (general memory). Their studies showed that gratitude journaling leads to enhanced mood, reduced psychological distress, and improved outlook, establishing its utility as a mental health intervention [17]. Similarly, Konopack and McAuley [21] suggests that spirituality and physical activity are partially mediated by self-efficacy perceptions, which influence adults’ quality of life. However, many of these studies are outdated, and there is a notable lack of research addressing both the physical and psychological aspects of positive psychological interventions, especially in racially diverse populations. Mind-body techniques, such as gratitude journaling, offer strategies for mitigating stress, promoting positive health behaviors, and potentially improving inflammatory biomarkers among Black BC survivors [15, 19, 22]. Despite evidence supporting the effectiveness of gratitude interventions in other populations, there remains a critical gap in research concerning their applicability and efficacy, specifically among Black women with breast cancer.

In this pilot study, we sought to address the multifaceted health needs of Black women with breast cancer by integrating culturally sensitive approaches into our intervention framework, guided by Giscombe’s Superwoman Schema and Fredrickson’s Broaden-and-Build theory [20, 23]. While general memory journaling has been shown to provide some mental health benefits, its limitations in directly addressing psychosocial outcomes in cancer survivors, particularly Black women with breast cancer, have not been fully explored. Gratitude journaling, by contrast, has demonstrated advantages in enhancing spiritual well-being and self-efficacy, key outcomes for this population [17–19]. Our study aims to fill this gap in knowledge by examining the associations of gratitude and perceived stress with spiritual well-being, exercise self-efficacy, and inflammatory biomarkers. We hypothesized that culturally tailored interventions would significantly enhance spiritual well-being among Black breast cancer survivors, laying the groundwork for future randomized controlled trials to further evaluate the efficacy of gratitude-based interventions in this underserved population.

## Methods

### Aim and design

This pilot study aimed to assess the feasibility and acceptability of a gratitude journaling intervention, with a primary focus on its feasibility and participant satisfaction. Additionally, the study sought to evaluate the preliminary efficacy of the intervention on various secondary outcomes, including dispositional gratitude, spiritual well-being, perceived stress, dimensions of the Superwoman Schema, exercise self-efficacy, and inflammatory biomarkers, among Black women with breast cancer. We employed a two-group, parallel random-assignment experimental design to compare a gratitude journaling intervention with a general memory journaling (attention control) group. The study protocol was registered prospectively with ClinicalTrials.gov (NCT05473026). A CONSORT checklist has been completed and included with this manuscript to ensure comprehensive reporting of the randomized controlled trial’s design and methodology in supplementary materials. The study followed a pre-post design, with outcomes measured at baseline and after eight weeks of intervention.

### Setting

Participants’ surveys and biospecimen collection were performed at the University of Florida (UF) Clinical and Translational Science Institute’s Clinical Research Center (CRC). The CRC offers comprehensive services for research studies encompassing a wide range of diseases and age groups and provides essential resources and support for data management and execution. The CRC includes a team of experienced research professionals, including registered nurses, research coordinators, dietitians, investigational pharmacists, and administrative personnel.

### Participants

The participants for the study were screened for eligibility via telephone. Eligible participants were English-speaking, self-reported African American or Black women with a history of breast cancer (Stage I–IV). Women on hormonal therapies and anti-human epithelial receptor 2 (HER-2) therapy were included. Exclusion criteria included self-reported regular meditation or gratitude practices (more than once a week for at least a month) or meeting the Centers for Disease Control and Prevention’s physical activity guidelines. Participants were compensated $100 within two time points for their participation.

### Sample size

In this feasibility study, the sample size was selected to evaluate key feasibility outcomes—such as recruitment rates, retention, and adherence—rather than to achieve statistical power for detecting treatment effects. A total of 26 Black female participants (13 per group) were recruited to allow for meaningful feasibility assessment and account for potential attrition. Although pilot study guidelines suggest a minimum of 12 participants per group to gather feasibility insights, we acknowledge that using a statistical formula or software, such as GPower, would provide a more precise approach based on anticipated effect sizes [24]. In future larger-scale randomized controlled trials, we plan to use GPower or a similar tool to calculate an appropriate sample size tailored to achieve statistical power for primary outcomes.

### Approval and consent

The pilot study (Protocol ID: 202201483) was approved by the Institutional Review Board (IRB) at the University of Florida. IRB approval was obtained before participant recruitment. Potential participants underwent a brief phone screening to assess their suitability for the study. During this screening process, they were provided with a concise overview of the study procedures, which included information regarding the nature of the questionnaires, blood-draw collection, and any associated risks. Upon signifying their understanding of the study procedures and risks, participants provided informed consent before enrollment.

### Recruitment

Participants were recruited using organic social media recruitment, flyers, and multiple specialized strategies from various resources, including the OAIC Pepper Center Clinical Research Core - Claude D. Pepper Center Participant Registry (IRB201601352), UF Health Cancer Center Community-Partnered Cancer Disparities Research Collaborative, UF Health Cancer Center Community Outreach and Engagement, UF Healthstreet, Integrated Data Repository, and UF Consent2Share Registries. Potential participants were screened for eligibility via telephone. Recruitment for the study began on November 8, 2022, marking the actual start of the study. Follow-up assessments were conducted at two key points: baseline, upon enrollment, and after eight weeks of intervention, with the final follow-up completed by July 25, 2023, which also marked the primary and overall completion date of the study. The study was conducted according to the planned timeline and reached its predefined sample size target. As a result, the trial ended on schedule, with no need for early termination. The adherence to the study protocol and the successful completion of follow-ups indicated that the study could provide meaningful preliminary data on the intervention’s feasibility, acceptability, and potential efficacy among the target population.

### Randomization and blinding

Before the appointment, participants were randomly assigned to either the gratitude journaling intervention group or the general memory journaling control group using a permuted block randomization method. The randomization sequence was computer-generated to ensure unbiased allocation. Participants were assigned to groups in a 1:1 ratio, ensuring equal distribution for a balanced comparison. Participants received concealed envelopes containing their group assignment and intervention details to maintain allocation concealment. These envelopes were prepared in advance and opened by the principal investigator during the initial appointment. The principal investigator then informed participants of their group assignment and provided instructions for the assigned intervention.

The study utilized a single-blind design, with blinding maintained for the research assistant responsible for collecting outcome data to minimize assessment bias. While the biostatistician was initially blinded during data analysis to reduce potential analysis bias, we acknowledge that biostatistician blinding may not be essential to this study’s design. Blinding primarily focused on the research assistant who collected outcome data to ensure that the individual assessing the outcomes was unaware of the participants’ group assignments, thereby enhancing the study’s internal validity.

### Intervention implementation

The intervention was designed as a self-directed journaling activity, so clinical therapists were not required to conduct it. Instead, the Principal Investigator, a board-certified nurse practitioner with clinical training, developed the intervention protocol. Research staff implemented the intervention, following a detailed protocol to ensure consistency and fidelity. Study staff received training in standardized journaling education and participant guidance to ensure uniformity across both gratitude and memory journaling groups. This structured protocol and staff training ensured fidelity without requiring clinical therapists.

### Gratitude journaling intervention group

The proposed study was built on the McCullough, Emmons [17] original gratitude journaling intervention to reduce the burden of journaling for breast cancer survivors. Participants received a gratitude journal and were instructed to journal at least twice weekly over an eight-week period. They were instructed to complete the following journaling prompt at least twice a week for 10–15 min: “There are many things in our lives, both large and small, that we might be grateful about. Think back over the day and write down on the line below all that you are grateful for today” (maximum six reasons). An educational goal-setting component focusing on physical activity was integrated into the gratitude journaling intervention, guided by the American Cancer Society’s Physical Activity Guidelines. At the start of the intervention (pre-intervention), participants were encouraged to set individualized physical activity goals, such as increasing daily steps or engaging in moderate-intensity exercises multiple times per week. Modules provided structured guidance on developing and adapting these goals, allowing participants to select the sequence and pace. At the end of the intervention (post-intervention), participants were asked to review their physical activity progress relative to their initial goals. This post-intervention assessment allowed participants to reflect on their physical activity achievements, challenges, and motivation levels. This physical activity component was designed to complement gratitude journaling, enhancing both physical and psychological well-being through a combined focus on movement and mindful reflection.

### General memory journaling control group

In the attention control group, participants engaged in general memory journaling as a neutral comparison to the gratitude journaling intervention. Participants were asked to document notable daily activities and reflections at least twice weekly for no more than 15 min per session. They received the prompt: ‘What are some memorable events that happened to you today, big or small (up to six memories)? Write a brief statement about it.’ Journals from both groups were collected at the end of the 8-week intervention to assess treatment fidelity.

### Data and biomarker collection

After screening for eligibility and obtaining verbal consent, participants were scheduled for an in-person appointment to collect baseline and post-intervention survey data, including collecting a blood sample over two-time points. Participants completed demographic and cancer history questionnaires and self-report instruments at each appointment. Informed written consent was obtained during the baseline appointment.

### Inflammatory biomarker collection

The collected blood samples were processed and stored in collaboration with the OAIC Metabolism and Translational Science Core at the UF Clinical Research Center. After blood sample collection, the plasma was separated, aliquoted, and stored at − 80 °C for later assay. A custom multiplex immunoassay panel for TNF-α, IL-6, and GDF15 (R&D Systems, Minneapolis, MN) was analyzed using a MILLIPLEX^®^ Analyzer 4.3 xPONENT System (Luminex Corp, Austin, TX). CRP levels were measured via ELISA (R&D Systems) utilizing BioTek Instruments (Winooski, VT) Synergy™ HTX Multi-Mode Microplate Reader and analyzed via Gen5 Microplate Reader Software (Bio-Tek Instruments). Analyses demonstrated a minimal interassay coefficient of variation < 14%.

### Assessment of feasibility and acceptability

The research assistant inputted various metrics using spreadsheet software to assess the feasibility and acceptability of the intervention, including recruitment and retention rates, participant adherence, and satisfaction measures. Recruitment and retention rates were tracked by monitoring the number of potential participants screened and enrolled in the study. The enrollment rate was calculated as the number enrolled relative to the total number of individuals screened to indicate the effectiveness of the recruitment process.

Participant adherence to the intervention was evaluated by analyzing journal logs to determine the frequency and consistency of engagement with the assigned journaling tasks. Adherence rates were calculated based on the number of completed journal entries relative to the expected frequency, indicating participants’ compliance with the intervention protocol. The proportions of women meeting the inclusion criteria relative to the number approached was calculated, with feasibility defined as > 75% of women approaching meeting the criteria. Additionally, the percentage of participants completing baseline assessment measures was computed, with feasibility defined as > 80% of participants completing these measures. Furthermore, the percentage of participants completing both baseline and post-intervention assessments was determined, with feasibility defined as > 80% of those completing baseline assessments completing post-intervention assessments.

### Demographic, psychosocial, and inflammatory biomarker measures

Demographic, psychosocial variables, and inflammatory biomarkers were measured at baseline (T1) and postintervention at eight weeks (T2).

#### Demographics (T1)

Demographic information, including age, sex, education, marital status, and body mass index was collected from participants at baseline (T1). Additional variables related to cancer and its treatment were recorded, such as time since diagnosis, cancer stage, grade, hormonal and HER2 + status, surgical type, chemotherapy, radiotherapy, and other relevant treatment modalities.

#### Psychosocial variables (T1, T2)

##### Dispositional gratitude

Dispositional gratitude was assessed using the Gratitude Questionnaire-6 (GQ-6) at baseline (T1) and follow-up (T2). The GQ-6 is a well-validated, self-administered 6-item scale with a Cronbach’s alpha of 0.82, indicating good internal consistency that measures four facets of dispositional gratitude, including intensity, frequency, span, and density. Participants rated each item on a 7-point Likert-type scale ranging from 1 (strongly disagree) to 7 (strongly agree) [17]. Scores range from 6 to 42, with higher scores implying a greater level of dispositional gratitude and lower scores indicating a decreased disposition in gratitude.

##### Spiritual well-being

Spiritual well-being was evaluated using the Functional Assessment of Chronic Illness Therapy - Spiritual Well-Being 12 Item Scale (FACITsp12), with a reported Cronbach’s alpha of 0.89, suggesting high reliability at both baseline (T1) and follow-up (T2). The FACITsp12 consists of 12 items rated on a Likert-type scale ranging from 0 (not at all) to 4 (very much), that assesses spiritual well-being over the past seven days among individuals with chronic illness [26]. Higher scores indicate enhanced spiritual well-being.

##### Perceived stress

Perceived stress levels were measured using the Perceived Stress Scale (PSS), with a Cronbach’s alpha ranging from 0.75, indicating strong internal consistency at baseline (T1) and follow-up (T2). The PSS comprises ten items rated on a 5-point Likert scale ranging from 0 (never) to 4 (very often), reflecting perceived stress experienced during the last month [27]. Higher scores indicate more perceived stress during the last month.

##### Superwoman schema

The Giscombe Superwoman Schema Questionnaire (G-SWS-Q) was utilized to assess the superwoman schema, including racial and gender identity, at both baseline (T1) and follow-up (T2). The G-SWS-Q has demonstrated high internal consistency, with subscale reliabilities ranging from 0.70 to 0.90. The G-SWS-Q consists of 35 statements rated from 0 (not true) to 4 (true all the time) across five subscales: (1) obligation to present an image of strength, (2) obligation to suppress emotions, (3) resistance to being vulnerable, (4) intense motivation to succeed, and (5) obligation to help others [23]. Higher scores indicate higher agreement with the identity.

##### Exercise self-efficacy

Exercise self-efficacy was assessed using the Stage of Motivational Readiness for Physical Activity questionnaire, a five-item tool with high reliability (Cronbach’s alpha = 0.85) at baseline (T1) and follow-up (T2). This highly reliable instrument comprises five items and classifies individuals into the pre-contemplation, contemplation, preparation, action, or maintenance stage of exercise behavior change based on the selection by the participant [28].

#### Inflammatory biomarkers (T1, T2)

Levels of tumor necrosis factor alpha (TNF-α), interleukin 6 (IL-6), growth/differentiation Factor-15 (GDF15), and C-reactive Protein (CRP) were measured at baseline (T1) and follow-up (T2) using a Luminex panel for TNF-α, IL-6, and GDF15, and an enzyme-linked immunosorbent assay (ELISA) kit for CRP.

#### Feasibility and acceptability

Feasibility and acceptability were assessed using exit interviews and the Client Satisfaction Questionnaire (CSQ), both administered at the end of the study. The CSQ, a reliable and validated tool widely used in healthcare settings, evaluates intervention acceptability. It comprises 8 items rated on a 4-point Likert scale, yielding total scores between 8 and 32, with higher scores indicating greater satisfaction [25]. This scoring range provides context for interpreting participant satisfaction. Additionally, the CSQ provided insights specific to the gratitude exercises. Exit interviews included structured and open-ended questions to explore participants’ perceptions of the intervention’s content, value, structure, and potential burden. These insights contributed to evaluating the overall feasibility and acceptability of the intervention.

### Data analysis

The statistical analysis was conducted using R software (Version 4.3.2). For group comparisons on changes between baseline and post-test, effect sizes were calculated using Cohen’s d, following established guidelines for interpreting effect sizes in pilot studies. Results for continuous variables are presented as mean (standard deviation, SD), and categorical variables as frequencies and percentages. Baseline demographic characteristics were examined by groups. Participant satisfaction was assessed using total CSQ scores post-intervention to evaluate acceptability. To justify using nonparametric tests, normality tests were performed on outcome variables in both intervention and control groups. For variables deviating from normality, the Wilcoxon rank-sum test and Fisher’s exact test were applied to calculate *p*-values and estimate effect sizes (ES) for group comparisons on changes between baseline and post-test for psychosocial and inflammatory biomarkers. Cohen’s guidelines for effect sizes were adapted to ranges for easier interpretation: small (0.10–0.23), medium (0.24–0.36), and large (≥ 0.37) [29]. Results of normality tests are included to support the use of nonparametric analysis.

## Results

### Participant flow

**Fig. 1:**
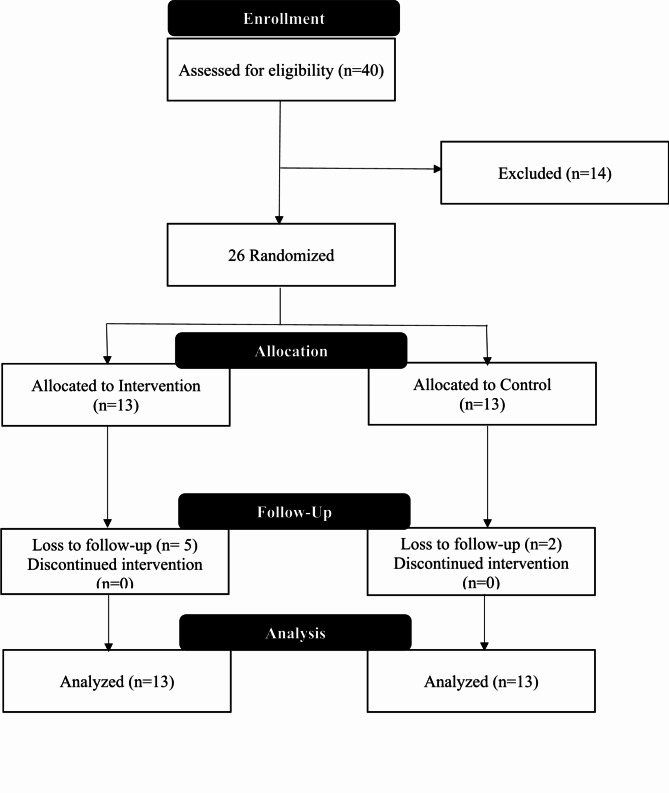
CONSORT Diagram

This study follows the CONSORT 2010 guidelines for reporting randomized trials [30]. The flow of participants through the study is illustrated in the CONSORT diagram (Fig. 1). Out of 40 individuals approached for participation, 26 met the inclusion criteria and were enrolled in the study. These participants were randomly assigned to either the gratitude journaling intervention group (*n* = 13) or the general memory journaling control group (*n* = 13). Eight participants completed the intervention in the gratitude journaling group, while five were lost to follow-up due to various reasons, including transportation and scheduling conflicts. In the control group, 11 participants completed the study, with two participants lost to follow-up. Overall, 73% of the participants (*n* = 19) completed both baseline and post-intervention assessments.

### Feasibility and acceptability

The feasibility analysis revealed that 65% of individuals approached met the inclusion criteria, and 26 participants completed baseline assessment measures with a completion rate of 100%. 73% of the participants (*N* = 19) completed baseline and post-intervention assessments. This slightly lower rate was attributable to transportation and work scheduling conflicts that some participants reported. Participants engaged in journaling activities regularly and, on average, three times per week, meeting the goal of at least twice a week. Regarding acceptability, participants reported high satisfaction with the intervention, as evidenced by a mean score of 29 on the CSQ-8.

### Analyses of demographic, psychosocial, and inflammatory biomarker outcomes

#### Demographics

Baseline demographics for the overall population (*N* = 26) and intervention groups are summarized in Table 1. At baseline, 26 participants were included in the study, with 13 assigned to either the control or intervention groups. The demographic characteristics and descriptive statistics in Table 2 showed no significant differences between the two groups. The mean age among participants was 65.0 years; gender and race distribution were equal across both groups, with all participants identifying as female Black breast cancer survivors (100.0%). Educational levels, employment status, and general area of residence also showed no significant differences between the groups.

**Table 1:** Demographics by groups at baseline (*N* = 26)

Characteristic	*N*	Overall (*N* = 26)	Control (*N* = 13)	Intervention (*N* = 13)	*p*-value
**Age**	26				0.396
Mean (SD)		65.0 (7.9)	66.7 (9.1)	63.4 (6.5)	
Median (25–75%)		64.0 (59.3 to 71.0)	67.0 (62.0 to 71.0)	63.0 (59.0 to 69.0)	
Range		51.0 to 83.0	51.0 o 83.0	52.0 to 72.0	
**Gender**	26				
Female		26 (100%)	13 (100%)	13 (100%)	
**Race**	26				
African American		26 (100%)	13 (100%)	13 (100%)	
**Hispanic**	26				
No		26 (100%)	13 (100%)	13 (100%)	
**Education**	26				> 0.999
Graduate degree		2 (7.7%)	1 (7.7%)	1 (7.7%)	
Some graduate school		2 (7.7%)	1 (7.7%)	1 (7.7%)	
College graduate		2 (7.7%)	1 (7.7%)	1 (7.7%)	
College 1–3 years		12 (46.2%)	6 (46.2%)	6 (46.2%)	
Grade 12 or GED		5 (19.2%)	2 (15.4%)	3 (23.1%)	
Some high school		3 (11.5%)	2 (15.4%)	1 (7.7%)	
**Employment**	26				> 0.999
Full-time		9 (34.6%)	4 (30.8%)	5 (38.5%)	
Part-time		3 (11.5%)	2 (15.4%)	2 (15.4%)	
Not working		14 (53.8%)	7 (53.8%)	7 (53.8%)	
**Income**	26				0.174
Less than $25,000		7 (26.9%)	6 (46.2%)	1 (7.7%)	
$25,000 to $49,999		9 (34.6%)	4 (30.8%)	5 (38.5%)	
$50,000 to $74,999		3 (11.5%)	2 (15.4%)	1 (7.7%)	
$75,000 or more		2 (7.7%)	0 (0.0%)	2 (15.4%)	
Prefer not to say		4 (15.4%)	1(7.7%)	3 (23.1%)	
**Relationship Status**	26				0.827
Single		16 (61.5%)	9 (69.2%)	7 (53.8%)	
Dating		2 (7.7%)	1 (7.7%)	1 (7.7%)	
Married		7 (26.9%)	3 (23.1%)	4 (30.8%)	
Prefer not to say		1 (3.8%)	0 (0.0%)	1 (7.7%)	
**Religion**	26				0.480
Christian		22 (84.6%)	11 (84.6%)	11 (84.6%)	
Not Religious		1 (3.8%)	0 (0.0%)	1 (7.7%)	
Other		2 (7.7%)	2 (15.4%)	0 (0.0%)	
Prefer not to say		1 (3.8%)	0 (0.0%)	1 (7.7%)	
**Weight**	26				0.137
Mean (SD)		201.8 (59.3)	181.1 (39.8)	222.5 (69.3)	
Median (25–75%)		186.5 (157.0 to 231.5)	180.0 (153.0 to 212.0)	205.0 (168.0 to 271.0)	
Range		120.0 to 352.0	120.0 to 250.0	136.0 to 352.0	
**Body Mass Index**	26				0.589
Mean (SD)		33.9 (8.7)	31.7 (5.7)	36.1 (10.7)	
Median (25%to 75%)		31.0 (28.0 to 39.3)	31.0 (28.0 to 36.0)	31.0 (27.0 to 44.0)	
Range		20.0 to 54.0	20.0 to 40.0	24.0 to 54.0	

* *p*-value were calculated using Wilcoxon rank sum test; Fisher’s exact test

**Table 2:** Change in psychological outcomes pre and post intervention by groups

Characteristic	Control (*N* = 11) Mean (SD)	Intervention (*N* = 8) Mean (SD)	*p*-value	Effect Size
Gratitude	-0.3 (1.0)	0.5 (1.3)	0.4	0.183
Spiritual Well-being	-1.8 (3.1)	3.6 (8.0)	0.014*	0.572
Perceived Stress	-1.3 (4.5)	-2.1 (4.9)	> 0.9	0.029
Superwoman Schema Subscale (Image of Strength)	-0.4 (3.9)	-0.4 (1.9)	> 0.9	0.010
Superwoman Schema Subscale (Suppress Emotions)	0.5 (5.2)	-0.8 (4.2)	0.3	0.239
Superwoman Schema Subscale (Resistance to Vulnerability)	-0.3 (7.6)	-2.1 (4.2)	0.4	0.190
Superwoman Schema Subscale (Motivation to succeed)	-1.0 (4.4)	-1.3 (3.2)	0.8	0.074
Superwoman Schema Subscale (Obligation to Help Others)	1.3 (6.8)	0.1 (5.5)	0.7	0.105
Superwoman Schema Total Score	1.1 (23.9)	-4.4 (11.4)	0.9	0.052
Exercise Self-Efficacy	-0.2 (0.6)	0.8 (1.5)	0.10	0.391

*p*-value were calculated using Wilcoxon rank sum test. The Wilcoxon effect size r is calculated as Z statistic divided by square root of the sample size (N): Z/√N\. * *p* < 0.05

#### Change in psychosocial outcomes pre and post-intervention

The study results showed a significant improvement in spiritual well-being (*p* < 0.05) post-intervention among participants in the intervention group compared to the control group (see Table 2). While other psychosocial outcomes showed positive trends from baseline to post-intervention, these were not statistically significant (see Table 2**)**. The intervention group showed a slightly higher mean change in dispositional gratitude (ES = 0.18, small). Spiritual well-being demonstrated a statistically significant improvement, with a large effect size (ES = 0.57). For the G-SWS-Q dimensions, the intervention led to a medium effect size for suppression of emotions (ES = 0.24) and a small effect size for willingness to expose vulnerability (ES = 0.19). Exercise self-efficacy displayed a marginally significant improvement with a medium effect size (ES = 0.391).

#### Change in inflammatory biomarker outcomes pre and post-intervention

Analysis of inflammatory biomarkers pre- and post-intervention indicated varying levels of response between the intervention and control groups (Table 3). For C-reactive protein (CRP), a small effect size (ES = 0.22) was observed, with the intervention group showing a mean increase of 3.1 mg/L (SD = 6.0) compared to an increase of 0.6 mg/L (SD = 2.6) in the control group. TNF-α also demonstrated a small effect size (ES = 0.18), with the intervention group exhibiting a greater mean decrease (-2.4 pg/mL, SD = 5.1) compared to the control group (-0.2 pg/mL, SD = 1.1). For IL-6 and GDF-15, effect sizes were minimal (ES = 0.01 and 0.12, respectively), with changes across groups not indicating significant trends. These findings suggest that while there were small changes in certain biomarkers, such as CRP and TNF-α, the overall impact of the intervention on inflammatory markers remained limited.

**Table 3:** Changes in Biomarker outcomes pre and post intervention by groups

Characteristic	Control (*N* = 11) Mean (SD)	Intervention (*N* = 7) Mean (SD)	*p*-value	Effect Size
C-Reactive Protein (mg/L)	0.6 (2.6)	3.1 (6.0)	0.4	0.224
Tumor Necrosis Factor Alpha (pg/mL)	-0.2 (1.1)	-2.4 (5.1)	0.5	0.182
Interleukin-6 (pg/mL)	0.0 (15.3)	3.0 (4.1)	> 0.9	0.011
Growth/Differentiation Factor 15 (pg/mL)	15.2 (696.4)	17.4 (89.4)	0.7	0.117

*p*-value were calculated using Wilcoxon rank sum test. The Wilcoxon effect size r is calculated as Z statistic divided by square root of the sample size (N): Z/√N. * *p* < 0.05

## Discussion

Breast cancer survivors, especially Black women, have been reported to encounter multifaceted challenges post-treatment, impacting their psychosocial and physical well-being [31]. Our study addressed this issue by investigating the efficacy of a gratitude journaling intervention tailored to Black women with BC while considering the intersectionality of race, gender, and cultural norms such as the Superwoman Schema. The observed improvement in spiritual well-being post-intervention highlights the potential of gratitude journaling to support holistic wellness among Black breast cancer survivors. However, as this study was designed to assess feasibility and acceptability, these findings should be interpreted as preliminary, with further investigation needed to evaluate efficacy. While no significant difference was found in dispositional gratitude between the intervention and control groups, the intervention group exhibited a trend towards increased gratitude, indicating a positive impact.

Historically, Black BC survivors have used spirituality to deal with hardships. Spiritual well-being is a primary coping mechanism during all phases of treatment and survivorship [32, 33]. In a psychometric study of the construct gratitude of 298 Blacks at risk for cardiovascular disease, Cousin, Redwine [34] found that dispositional gratitude correlates with spiritual well-being and positive affect. Moreover, gratitude and spiritual well-being are related to improved mood, higher rates of self-efficacy, and lower levels of inflammatory biomarkers in other clinical populations [18]. Similar research findings from gratitude studies among cancer survivors show that gratitude is associated with lower levels of psychological distress [19, 35, 36]. Yet, there is little evidence to support the impact of dispositional gratitude on perceived stress among Black women.

The Superwoman Schema dimensions reflect culturally ingrained behaviors among Black women that influence their health behaviors and outcomes [9]. In a study of Black women (*n* = 208) that examined whether the Superwoman Schema modified the relationship between racial discrimination and allostatic load (a marker of cumulative stress), researchers reported that two of the subscales, “maintaining unwavering strength” and “suppressing emotions,” were each protective factors, while “placing the need of others before themselves” exacerbated the health risk associated with experiencing racial discrimination [37]. In our study, while changes in Superwoman Schema dimensions were observed, they were not consistently significant across all domains, suggesting potential variability in participants’ responses to the intervention. However, our intervention appeared to influence participants’ tendencies related to emotional suppression and vulnerability, indicating a potential shift in coping strategies and self-perception. A lack of significant changes in other dimensions suggests a complex relationship between cultural norms and health behaviors. Future interventions may benefit from further exploration of these dimensions to better understand their implications for health outcomes among Black women with BC.

Most gratitude interventions utilized across the cancer continuum only focused on their impact on psychological outcomes and cognitive measures of their work, while few have examined inflammatory biomarkers. Higher inflammatory biomarkers and their reactivity to stress may predict worsening outcomes among Black BC survivors. For example, Black BC survivors have higher serum IL-6 than White women, suggesting an additional risk of BC complications and poor prognosis [38]. Although more evidence is needed, gratitude, positive affect, self-efficacy, and spiritual well-being have been linked to improved inflammatory biomarkers [15, 18, 39]. While some inflammatory biomarkers exhibited changes indicative of potential improvements in inflammation levels, the overall impact did not reach statistical significance across all biomarkers examined. These findings suggest that while the intervention may have influenced certain aspects of participants’ health, further research is needed to elucidate its full effects on inflammatory processes.

Exercise self-efficacy, a crucial determinant of physical activity engagement, displayed marginal improvement post-intervention, indicating the intervention’s potential to enhance participants’ confidence in adopting healthier lifestyle behaviors. Given the importance of physical activity in mitigating BC-related complications, this finding underscores the holistic approach of gratitude interventions in promoting overall well-being. Individuals with BC and high exercise self-efficacy are more likely to initiate and maintain physical activity, overcome barriers, and persevere through challenges. Conversely, low exercise self-efficacy may hinder engagement in exercise programs, leading to decreased adherence and limited health benefits [40, 41]. By addressing exercise self-efficacy, healthcare professionals, support groups, and community organizations can empower BC survivors to embrace physical activity as an integral part of their post-treatment journey. Lastly, the intervention’s high feasibility and acceptability underscore its relevance and suitability for the target population. Despite logistical challenges affecting post-intervention assessment completion, participants demonstrated strong adherence to journaling activities, indicating a high level of engagement and commitment.

## Limitations and future directions

Several limitations warrant consideration, including the short intervention duration, control group design, and potential confounding variables. The eight-week intervention may have been too short to observe long-term changes in psychosocial and inflammatory outcomes, potentially leading to imprecision in estimating the sustained effects of the gratitude intervention. Additionally, the use of an attention control group may have impacted the effect size observed in this study. In clinical psychology, attention control groups can sometimes produce notable changes in outcomes, potentially reducing the observable difference between the intervention and control groups. Future studies may consider alternative control designs, such as a wait-list or no-treatment group, to better capture the intervention’s impact. Finally, exploring the mechanisms underlying the observed changes in psychosocial and inflammatory outcomes will be essential for developing more targeted and effective interventions in future research.

## Conclusion

Gratitude journaling intervention for Black women breast cancer survivors has yielded significant findings regarding both feasibility and the intervention’s impact. Findings from this study demonstrated the applicability and high acceptability of within this population. Participants reported high satisfaction levels with the intervention, underscoring its usability in addressing the psychosocial needs of Black women breast cancer survivors.

Significant findings emerged from the study, highlighting the potential efficacy of gratitude journaling in enhancing spiritual well-being among participants. While no significant differences were observed in dispositional gratitude between the intervention and control groups, the intervention demonstrated a notable improvement in spiritual well-being post-intervention, indicating its effectiveness in fostering holistic wellness among survivors. Promising trends in exercise self-efficacy were observed, suggesting that gratitude journaling may empower survivors to adopt and maintain healthy lifestyle behaviors. Future research should focus on scaling up interventions with a larger sample size tailored to meet BC survivors’ diverse needs. Gratitude journaling is a valuable tool in empowering and uplifting BC survivors to promote resilience and enhance overall well-being during their survivorship journey.

## Electronic supplementary material

Below is the link to the electronic supplementary material.

Supplementary Material 1

## Data Availability

The datasets generated and analyzed during the current study are available from the corresponding author [LC] upon reasonable request.

## Abbreviations

BC: Breast cancer
FACIT-Sp12: Functional assessment of chronic illness therapy - spiritual well-being 12 item scale
GQ-6: Gratitude questionnaire-6
PSS: Perceived stress scale
TNF-α: Tumor necrosis factor-alpha
IL-6: Interleukin-6
CRP: C-reactive Protein
GDF15: Growth/differentiation factor-15
CSQ-8: Client satisfaction questionnaire
G-SWS-Q: Giscombe superwoman schema questionnaire
UF: University of florida
CRC: Clinical research center
IRB: Institutional review board
OAIC: Older americans independence center
ELISA: Enzyme-linked immunosorbent assay
ES: Effect size
